# Do Infrared Thermometers Hold Promise for an Effective Early Warning System for Emerging Respiratory Infectious Diseases?

**DOI:** 10.2196/42548

**Published:** 2023-05-03

**Authors:** Rui Li, Mingwang Shen, Hanting Liu, Lu Bai, Lei Zhang

**Affiliations:** 1 China-Australia Joint Research Center for Infectious Diseases School of Public Health Xi'an Jiaotong University Health Science Center Xi'an China; 2 Melbourne Sexual Health Centre Alfred Health Melbourne Australia; 3 Central Clinical School Faculty of Medicine, Nursing and Health Sciences Monash University Melbourne Australia

**Keywords:** respiratory infectious diseases, early warning, infrared thermometer, theoretical framework, economic burden, outbreak prevention, warning system, community health, infectious disease, smartphone device, digital health surveillance

## Abstract

**Background:**

Major respiratory infectious diseases, such as influenza, SARS-CoV, and SARS-CoV-2, have caused historic global pandemics with severe disease and economic burdens. Early warning and timely intervention are key to suppress such outbreaks.

**Objective:**

We propose a theoretical framework for a community-based early warning (EWS) system that will proactively detect temperature abnormalities in the community based on a collective network of infrared thermometer–enabled smartphone devices.

**Methods:**

We developed a framework for a community-based EWS and demonstrated its operation with a schematic flowchart. We emphasize the potential feasibility of the EWS and potential obstacles.

**Results:**

Overall, the framework uses advanced artificial intelligence (AI) technology on cloud computing platforms to identify the probability of an outbreak in a timely manner. It hinges on the detection of geospatial temperature abnormalities in the community based on mass data collection, cloud-based computing and analysis, decision-making, and feedback. The EWS may be feasible for implementation considering its public acceptance, technical practicality, and value for money. However, it is important that the proposed framework work in parallel or in combination with other early warning mechanisms due to a relatively long initial model training process.

**Conclusions:**

The framework, if implemented, may provide an important tool for important decisions for early prevention and control of respiratory diseases for health stakeholders.

## Introduction

Major respiratory infectious diseases, such as influenza, SARS-CoV, and SARS-CoV-2, have caused historic global pandemics with severe disease and economic burdens. The 1918 influenza pandemic that killed 50 to 100 million people globally is the single deadliest catastrophe in recent human history [1]. By contrast, the recent COVID-19 pandemic has caused 676 million infections and 6.9 million deaths, and multiple COVID-19 variants are still rampant across the world [2]. One common characteristic of these viruses is their high transmissibility, often leading to exponential growth in the early phase of the outbreak, before any public health interventions are initiated. They are also characterized by a large number of individuals with clinical symptoms, such as fever, cough, and sore throat. However, primary care is often unprepared for a sudden surge of infections with unknown etiology during the early outbreak. The optimal timing for public health intervention is already missed before hospitals detect a large influx of patients. Early warning and timely intervention are key to suppress such outbreaks. Hence, an effective early warning system would provide stakeholders with timely and crucial information that characterizes the outbreak and enables proportional prevention and intervention. Major early warning systems (EWSs) for infectious diseases have been established across continents, such as the Early Warning Alert and Response Network of the World Health Organization (WHO), the National Notifiable Diseases Surveillance System of the United States, the European Surveillance System, and the National Disease Report System of China. However, all these institutions are hospital-based, with surveillance focused on only known infectious diseases.

## Community-Based EWS for Respiratory Diseases

**Figure 1:**
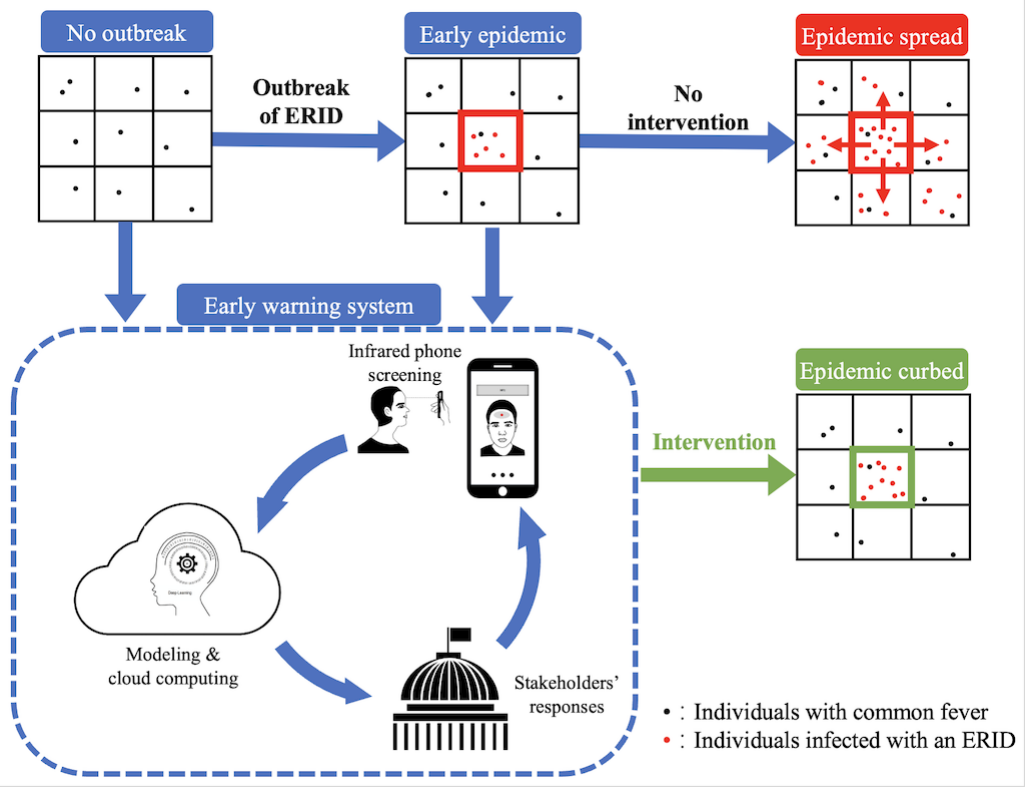
Schematic flowchart of a community early warning system based on infrared thermometer–enabled mobile phone screening and a big data platform for the early prevention of ERIDs. ERID: emerging respiratory infectious disease.

We propose a theoretical framework for a community-based EWS that will proactively detect temperature abnormalities in the community based on a collective network of infrared thermometer–enabled smartphone devices. Overall, the framework uses advanced artificial intelligence (AI) technology on cloud computing platforms to identify the probability of an outbreak (Figure 1). It hinges on the detection of geospatial temperature abnormalities in the community based on mass data collection, cloud-based computing and analysis, decision-making, and feedback. First, individuals proactively self-determine their body temperature using a smartphone equipped with an infrared thermometer or linked to a Bluetooth-enabled infrared thermometer. Most recently, Huawei has pioneered the development of an infrared thermometer–equipped smartphone in its latest model (the Honor Play 4 Pro). Mass body temperature data of individuals in the community can be collected and integrated into a cloud-based data platform with users’ consent. Second, an AI-driven algorithm on the cloud-based computing platform is used to process the information and determine the probability of an outbreak based on historical patterns. This relies on the establishment of a background incidence of temperature abnormalities that calibrates the scenario without an outbreak. AI-based computing approaches such as machine learning for geolocation [3] are capable of identifying irregularities in complex geospatial patterns of body temperature distribution. Based on this, the algorithm quantifies the probability and severity of a potential outbreak. Third, the EWS performs real-time communication with stakeholders, such as juridical centers for disease control, and prompts stakeholders to authenticate the early warning signal by triangulating the information with reports from other surveillance methods and hospitals. This enables stakeholders to act promptly and decisively to introduce emergency plans in response to the outbreak. Consequently, measured restrictions in proportion to outbreak severity can target the affected community to reduce the mobility of the population and implement nonpharmaceutical interventions.

## Feasibility of the EWS

The EWS may be feasible for implementation considering its public acceptance, technical practicality, and value for money. First, infrared thermometers have been widely accepted and used for COVID-19 prevention and control globally. Compared with conventional mercury and electronic thermometers, they are convenient, with a very short operation time, and are portable. Their recent integration with smartphones provides a strong advantage for users to access body temperature monitoring given their convenience and accessibility. With users’ consent, body temperature information may be collected on a centralized data platform through a mobile phone app. Second, the technologies required for the EWS are readily available. The use of infrared thermometers for fever detection is a mature technology, with at least 80% sensitivity and 95% specificity [4,5]. Recent advancements in 5G data transmission enable real-time data transmission, integration, and result responses. Cloud-based services, such as Amazon, Microsoft, and Alibaba, provide extensive commercial cloud data storage space and computing capacity. Third, the cost of implementing the EWS is low. As infrared thermometers have increasingly become a standard feature on smartphones, the willingness-to-pay threshold for end users has been substantially reduced. Even for smartphones with no infrared thermometer, external Bluetooth-enabled infrared thermometers are available at a low cost. With the availability of cloud data storage and computing and the reduction in their cost, the cost of hardware establishment, operation, and security maintenance are all substantially reduced for end users.

## Obstacles to the EWS

Several obstacles to establishing the EWS remain. First, the accuracy of infrared thermometers can be strongly affected by the surrounding environment. Blazing sun, cold wind, and hot indoor temperatures can all bias their measurements. Friendly tips to users to remind them to choose a relatively stable environment for temperature detection may be necessary. Second, the detection of temperature abnormalities in a community is accurate only when a sufficient number of users agree to upload their data, indicating that high public acceptance is a requirement. This is related to timely feedback and a guarantee of data privacy for such large-scale data collection. However, the current COVID-19 pandemic has prepared ordinary people to be more cautious about respiratory diseases and open to the idea of early warning. There is an urgent need to start a pilot community. Third, the determination of the cutoff for temperature abnormalities for public health intervention will require abundant historical data and repeated training of the model. This means a relatively long initial model training process. Therefore, it is important that the proposed framework work in parallel or in combination with other early warning mechanisms, such as the Early Warning Alert and Response Network of the WHO, the National Notifiable Diseases Surveillance System of the United States, the European Surveillance System, and the National Disease Report System of China. Finally, it requires stakeholders’ judgment to weigh the risk of a substantial disease outbreak, the economic implications, and societal considerations to make a balanced intervention decision.

In conclusion, we propose an innovative and practical framework for a community-based EWS for ongoing and future outbreaks of emerging respiratory infectious diseases. The framework, if implemented, may provide an important tool for important decisions for early prevention and control of respiratory diseases for health stakeholders.

## Abbreviations

AI: artificial intelligence
EWS: early warning system
WHO: World Health Organization
